# Cognitive and plastic recurrent neural network clock model for the judgment of time and its variations

**DOI:** 10.1038/s41598-023-30894-4

**Published:** 2023-03-08

**Authors:** Quentin Hallez, Martial Mermillod, Sylvie Droit-Volet

**Affiliations:** 1 Université Lumière Lyon 2, Laboratoire Développement, Individu, Processus, Handicap, Éducation (DIPHE), 5 Avenue Pierre Mendès France, 69500 Bron, France; 2 Univ. Grenoble Alpes, Univ. Savoie Mont Blanc, CNRS, LPNC, 38000 Grenoble, France; 3 Université Clermont Auvergne, CNRS, LAPSCO, Clermont-Ferrand, France

**Keywords:** Human behaviour, Computational science

## Abstract

The aim of this study in the field of computational neurosciences was to simulate and predict inter-individual variability in time judgements with different neuropsychological properties. We propose and test a Simple Recurrent Neural Network-based clock model that is able to account for inter-individual variability in time judgment by adding four new components into the clock system: the first relates to the plasticity of the neural system, the second to the attention allocated to time, the third to the memory of duration, and the fourth to the learning of duration by iteration. A simulation with this model explored its fit with participants’ time estimates in a temporal reproduction task undertaken by both children and adults, whose varied cognitive abilities were assessed with neuropsychological tests. The simulation successfully predicted 90% of temporal errors. Our Cognitive and Plastic RNN-Clock model (CP-RNN-Clock), that takes into account the interference arising from a clock system grounded in cognition, was thus validated.

## Introduction

For decades there has been a consensus that humans are equipped with an internal clock system for accurate time measurement^1,2^. However, despite having this internal clock system, evidence has accumulated that humans vary in their estimation of time and are prone to the under- and over-estimation of time. These misjudgments of time have been found in particular among individuals with limited cognitive abilities, such as young children^3,4^. Numerous neuropsychological studies which have assessed the cognitive abilities of children of different ages have shown that misjudgments of time increase in line with a decrease in children's abilities, mainly in terms of working memory and attention^5–8^. In accordance with internal clock models^1,9,10^, researchers have attributed these misjudgments of time to cognitive modules added to the clock module as part of a wider temporal information processing. For example, time distortions have been explained by the time units emitted by the clock not being entered into a person’s memory due to a lack of attention allocated to time^11^ (i.e., the attention hypothesis). They have also been explained by the loss of units in memory when the retention interval increases^12,13^ (i.e., the memory hypothesis), and by a noisy representation of the standard duration in reference memory due to a less efficient learning process^14,15^ (i.e., the learning by iteration hypothesis). The reliability of the internal clock system itself has not, therefore, been called into question.

The idea behind the internal clock models is that, for the measurement of time, the brain would be able to automatically extract the temporal properties of neurons that generate oscillatory activity fluctuating from negative to positive states, linked to their action potential. It was initially suggested that the internal clock could operate with a single oscillator, in the pacemaker models^9,10,16^. Nonetheless, these models also include a counting system (accumulator) because the neuron would make several revolutions during the duration to be estimated. The so-called pacemaker-accumulator models have been extremely influential in the psychological timing literature^2^. However, they seem difficult to apply from a neurobiological point of view. Inspired by Miall’s work^17^, Matell and Meck with their Striatal Beat Frequency Model^18^ (SBF) then came up with the idea that, if the brain is based on various neurons that oscillate at different frequencies, then it would take many revolutions for the neurons to return to common states, thus making is possible to process both short and long durations, without any counting system. At the beginning of a duration to be estimated, the brain would base its judgment on neurons which have just discharged, and would retain the "signature" specific to the duration by recording the state of oscillation of the neurons. Timing could be carried by linear decoders trained to recognize states of a neural network^19^.

Recently, Recurrent Neural Networks (RNN) models have successfully simulated the processing of time through neural oscillatory functioning^20–24^. In the current study context of computational neurosciences, we therefore employed a RNN model based on a bank of neurons (see Chapter 2 Model 1, RNN). We did so for three reasons. First, the RNN model is easier to understand with regard to the basic biological properties of the top-down synaptic connectivity of humans^25^. In top-down processing, perceptions are strongly influenced by cognitive skills that differ between individuals, e.g., prior knowledge^26^. For example, neurons in the motor cortex increase their synchrony when animals are trained to expect a 'go' signal^27^. Second, time perception has been shown to involve top-down processes^28^, being also influenced by expectations and prior knowledge^29,30^. Third, the recurrent networks have already proved efficient for a wide range of dynamical phenomena such as facial recognition^31^ and serial recall^32^. The aim of our study was thus to use the RNN and modify its functioning to simulate the time processing of individuals (i.e., children as well as adults) with various cognitive capacities.

In our study, we therefore tested a RNN model to replicate the oscillatory brain activity involved in time processing with top-down connection for anticipation and prediction processes. However, our originality was to add four new parameters to this model, to allow us to account for inter-individual differences in time judgment (see Chapter 3). In this new model, we added a first parameter enabling consideration of the possibility of a “fallible” clock system. The “fallible” physiological aspect of the clock system can be conceived in terms of neural plasticity. The brain system remains plastic throughout life to allow individuals to learn^33^. Young children learn quickly, often even faster than adults, which suggests that their clock system would be highly plastic. However, although greater plasticity promotes learning, it also increases the probability of making critical mistakes due to forgetfulness^34^. As is explained in more detail below, the clock plasticity is considered in the RNN model by modulating the “Fahlman offset”^35^. The Fahlman offset, which involves adding a small constant number to the derivative of the sigmoid function so that it does not go to zero for any output value, has proven to be a simple and efficient way to simulate plasticity loss^34^. For example, adding a constant of 0.1 to the sigmoid function before using it to scale the error prevents neuron values from approaching 0 or 1, and avoids the flat spots in the sigmoid function where the synaptic weights can become entrenched.

In our plastic and cognitive version of the RNN-Clock model, we also added three cognitive parameters based on the results of prior developmental studies which have shown that attention, working memory, and learning for temporal reference memory explain a large proportion of inter-individual differences in temporal judgments^3,4^. As we discuss later, attention was considered in our model by changing the amount of information that could be selected (more or fewer values in the database), varying iterative learning by modulating the number of epochs^36^, and varying memory by modulating the number of formal neurons embedded in the hidden layer^37^.

In summary, in our computational modeling approach, we created three versions of the same model allowing us to simulate participants’ data in a reproduction task. First, we tested a Simple Recurrent Neural Network (RNN) in order to replicate the Recurrent Neural Network model used in the judgment^18,20^. Second, we launched this model multiple times by varying each of the four parameters described above: (1) clock plasticity; (2) attention; (3) temporal learning by iteration; and (4) memory, one by one, in turn. For each simulation we calculated the temporal dispersion coefficient, which was the slope calculated using a linear regression associated with the cumulated Euclidean distance of the error generated by each of the models. Third, we gathered all these temporal dispersion coefficients into a second model, which was a MLP (Multilayer Perceptron) that could hold all the errors of the RNN associated with the different modulations of the four parameters. Fourth, we created a third and final version of our model in order to compare the outputs of that final model with the data collected from 308 human participants in a temporal reproduction task (both children and adults), whose individual cognitive capacities were assessed using neuropsychological tests. The final model used real individual neuropsychological test scores and age (inputs) to compute the state of each of the four parameters of our clock system, in order to predict individual errors in the temporal estimation for a temporal reproduction task (outputs).

## Model 1: recurrent neural network model

### Internal clock basis

**Figure 1:**
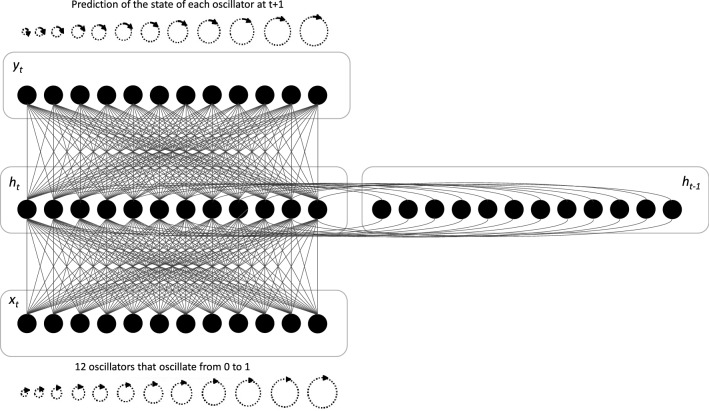
Representation of the Simple Recurrent Neural Network model, with *x*_*t*_, input vector of the 12 oscillators at *t0, ht*, hidden layer vector at time *t*; *x*_*t*+1_output vector; *A*, *R*,*U* parameters paired with each layer. The Figure was designed by the present authors.

We created a Simple Recurrent Neural Network (RNN) as the basis of our internal clock model. An RNN is an artificial neural network composed of four layers: an input layer (*x*_*t*_), a hidden layer (*h*_*t*_), a context layer (*h*_*t-*1_), and an output layer (*y*_*t*_) (see Fig. 1). The input layer (*x*_*t*_) works as a buffer, as the signal is not being transformed. The signal is transformed at the level of the hidden layer (h*t*) as units interact with each of the neurons of the prior structure by means of a non-linear sigmoid transfer function.

For each time *t*, the activity of the hidden layer (*h*_*t*_) was recorded in the context layer (*h*_*t−*1_). The context layer then re-injected the recorded signal in the next iteration *t* + *1*.

The hidden layer *h*_*t*_ was thus updated for each time *t* following Eq. (1):

1 $$h_{t} = \sigma \left( {Ax_{t} + Rh_{t - 1} } \right)$$

where *σ* is the sigmoid activation function applied coordinate wise, while *A* and *R* refer to the weight between each of the units. Finally, the output layer (*y*_*t*_) sums the signals fed to it according to Eq. (2):

2 $$y_{t} = f\left( {Uh_{t} } \right)$$

*U* is the weight assigned by the program for each of the units constituting the hidden layer, and *f* the soft-max function which converts a vector of real numbers into a probability distribution of possible outcomes. Nevertheless, the system does not find the weight of each of the neurons at one time. Calculation results from so-called gradient retro-propagation, which consists of updating the weight of each neuron from the last layer to the first^38^. It is therefore necessary to fix the number of allowed epochs. An epoch describes the number of times the algorithm sees the entire dataset. So, each time the algorithm has seen all samples in the dataset, an epoch is completed. Once the retro-propagation of the gradient is complete based on the number of epochs, we can then calculate the error between the output given by the network and the predicted vector of the output.

**Figure 2:**
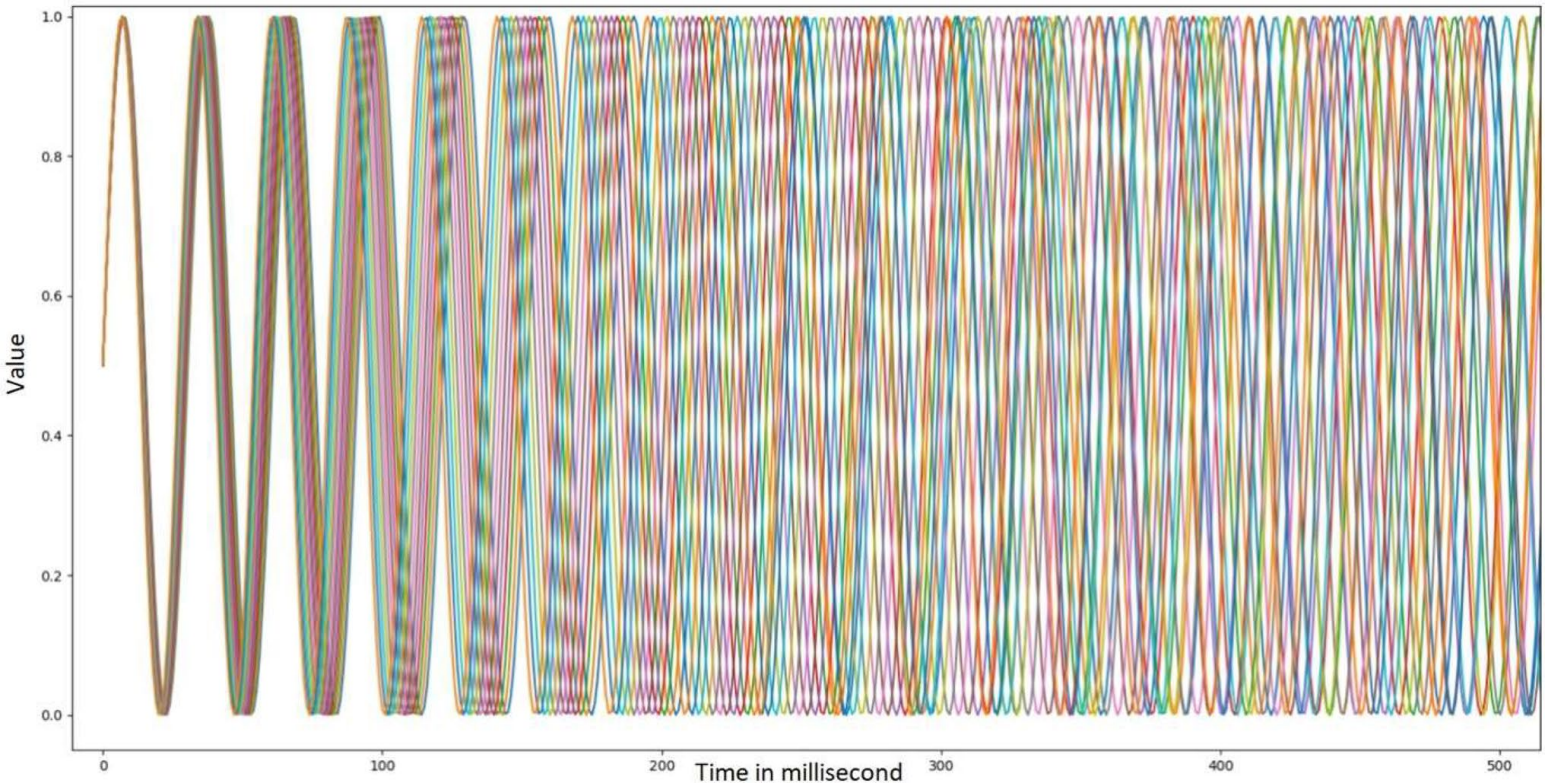
Representation of oscillation value for each oscillator.

Within our RNN, a total of 12 formal oscillatory neurons were inserted as input neurons, each one corresponding to a neuron fluctuating from negative to positive states every *t*_millisecond_. The respective values of the oscillators (see Fig. 2) were (Hz): *F*_*o*1_ = 32.9, *F*_*o*2_ = 33.2, *F*_*o*_3 = 33.6, *F*_*o*_4 = 34, *F*_*o*_5 = 34.3, *F*_*o*_6 = 34.7, *F*_*o*_7 = 35.1, *F*_*o*_8 = 35.5, *F*_*o*_9 = 35.9, *F*_*o*10_ = 36.3, *F*_*o*11_ = 36.8, *F*_*o*12_ = 37.2. These Hertz values were selected for their similarity to the pattern of neural gamma oscillations observed in humans^39^, which have been detected in the process of awakening^40^. In addition, in our model, such as in the SBF model, the selected neurons for the measurement of time were synchronized at the beginning of a stimulus to be timed. The 12 oscillators started at 0.5 for *t*_0_. Therefore, values lower and higher than 0.5 suggest a negative and a positive state, respectively. The model also creates an output of the recurrence, but its use is inherent to the model and its output value is not used later. The output of the model is therefore the prediction of the state of each of the 12 oscillators.

**Figure 3:**
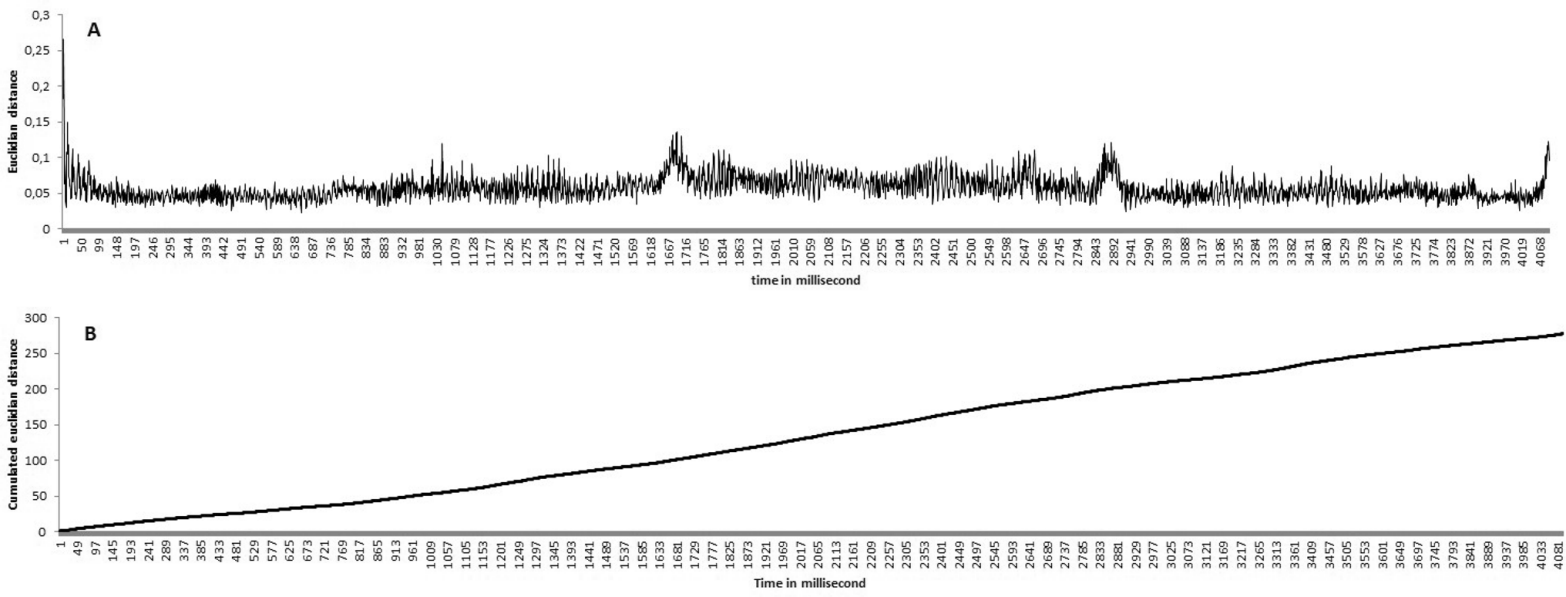
Illustration of (**A**) Euclidean distance and (**B**) cumulative Euclidean distance for each millisecond.

In other words, the inputs of the first model correspond to the oscillations holding a certain value which differs at each millisecond depending on their rates. Then, the model learns by backpropagation to find *t* + 1 based on *t* for each millisecond duration. Finally, the model tries to estimate a duration from the initial oscillation’s state by adding t + 1 (e.g., + 1 ms) until reaching the duration it must estimate. Predictions of oscillators’ states were thus made for every t_millisecond_. That being so, if a participant has to estimate exactly two seconds, then we are interested in the predictions based on the prediction of the oscillator’s state at t_2000_. This operating logic creates an error for every millisecond (Fig. 3A) accumulated (Fig. 3B), which is the cumulative average of the Euclidean distance averages of the objective—subjective differences. This also allows the model to reproduce the scalar properties of timing (e.g., an increase in error over time) characteristic of timing in human^2^.

In our model, the synaptic weights were adjusted by means of stochastic back-propagation. The momentum was fixed to 0.9 and the learning rate to 0.1. This learning rate parameter was chosen because it produces the fastest convergence^41^ and corresponds to the standard value of the learning rate generally used. In addition, in our simulation we fixed the standard back-propagation (number of epochs) to 500, the training to a maximum of 4096 ms, the Fahlman offset to 0.1, and the number of formal neurons in the hidden layer to 16, although we modulated the modalities of these variables later. Figure 3 shows the* t* values estimated from the model from 0 to 4096 ms for normal Euclidean distance (Fig. 3A) and cumulative Euclidean distance (Fig. 3B) for these specific parameters.

### Plastic-clock and cognitive components in the RNN clock model

The aim of our model was to grasp the greater time distortion and the greater temporal variability in participants with lower cognitive capacities (including children of different ages and adults). We therefore modulated a total of four components: plasticity, temporal learning by iteration, memory, and attention.

**Figure 4:**
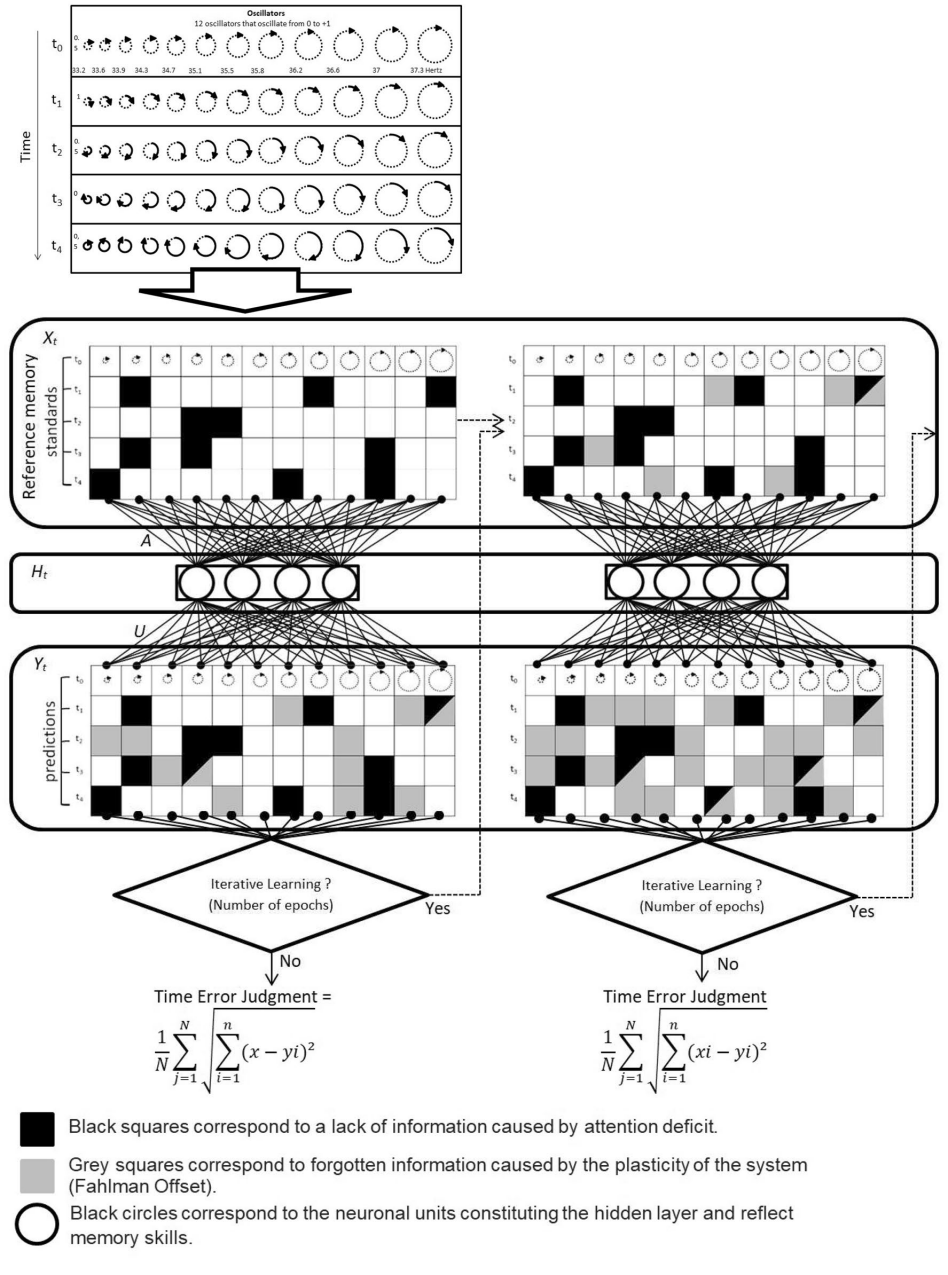
Simplified illustration of Cognitive and Plastic RNN clock model (CPC model). The first table shows the oscillation over time (from 0 to 1) of 12 neuronal oscillators. The oscillation information is more or less integrated according to subjects’ attentional capacities. An attention deficit generates a lack of information for a given time and a given oscillator (represented in the graph by black squares). The information held with respect to these oscillators is then combined into a memory system so it can generalise the anchored information. The information that springs out of this structure corresponds to the temporal estimation of the subject, to which gray squares have been added, corresponding to forgetfulness. The proportion of forgetfulness corresponds to plasticity (Fahlman offset), since a more plastic system learns faster but also forgets more easily. Finally, the subject can give an estimate which corresponds to the cumulative average of the Euclidean distance averages of the objective—subjective differences. Nevertheless, in the case of iterative temporal learning, the whole operation will be restarted. Further, predictions will increasingly suffer from the weight of the prior estimation, which will scramble the initial standard duration, thus increasing the proportion of noise in the reference memory.

With regard to attention, when participants have difficulty paying attention to time, their time estimates are noisier^6,7,42^. In our model, the *attention* parameter was located in the input layer, *X*_*t*_ and attention deficit results in a decrease in the amount of information presented in the database relating to the evolution of oscillators’ states according to the progress of *t* (as is illustrated in Fig. 4 by black squares). The system can therefore trace the evolution of oscillations up to 1024, 1792, 2560, 3328 or 4096 ms.

The *memory* parameter was located in the hidden layers, *H*_*t*_. We acted on the memory system by increasing or decreasing the retention capacity via changing the number of formal neurons within the hidden layers. Indeed, increasing the number of neurons increases the model’s capacity to remember what has previously been presented in the database^37^. We thus chose a varied number of neurons, i.e., 8, 16, 32, 64, and 128, in the hidden layer in order to model this variable. Furthermore, as was explained above, a plastic system leads to faster learning but also to further forgetting, thus corresponding to the loss by the subject of the state of oscillations. As plasticity is associated with forgetting, it occurs during the computation as illustrated in Fig. 4 via the grey squares on *Y*_*t*_. During the computing—that is, during the temporal processing—the subject may indeed lose track of the accumulated units if the plasticity is too high.

For the *clock plasticity*, as reported above, we acted on this *parameter* by changing the Fahlman offset^32^. The Fahlman offset index = 0.01, 0.015, 0.03, 0.06, 0.125, 0.25 and 0.5. In our neural networks model, the timer of the clock-like system is thus associated with a memory system, as has already been suggested by other authors^1,12^. Finally, the predictions join the *iterative learning* structure (see Fig. 4). It is at this level that the subject makes his/her judgment. Nevertheless, in the case of iterative temporal learning, the whole operation will restart, thus gradually increasing the proportion of noise in the temporal reference memory. This learning-by-iteration parameter involved in temporal reference was modulated by modifying the number of epochs^36^. As previously stated, an epoch corresponds to the number of times the algorithm sees the complete dataset. This factor has been linked with iterative learning, as less learning solicits less feedback and thereby fewer epochs. The selected epochs were 100, 200, 300, 400, 500.

The model was launched 875 times, modulating each parameter one by one (5 attention modalities × 5 memory modalities × 5 learning iteration × 7 plasticity = 875). Based on each of these computations the program predicted, with more or less error (e.g., Euclidean distance), the states of each of the 12 oscillators from 1 to 4095 ms. Based on the cumulated Euclidean distance of this error through time, we launched a linear regression in order to generate an error slope that we called a temporal dispersion coefficient, associated with each of the computations (all *ps* < 0.001; *R*^2^ ≥ 0.98). The link to OSF (section linear estimations) shows the matched dispersion coefficient with the different values of each parameter, i.e., for different numbers of neurons (memory parameter), different plasticity values (Fahlman offset), different epochs (learning parameter) and different amounts of information presented in the input database (attention parameter). In order to statistically analyse this influence, we calculated the mean error when we varied the values of a parameter, independently of other parameters. A first ANOVA was launched on the memory parameter, that is, the five neuron conditions (8, 16, 32, 64 and 128 neurons) inserted within the hidden layer. The ANOVA showed a significant main effect of this variable, *F*(4, 696) = 325.99, *p* < 0.001, *η*^2^_*p*_ = 0.65. As shown Table 1, additional paired t-test revealed that all the conditions differed from each other (Bonferroni, *ps* < 0.001), generating decreased errors as the number of neurons implemented in the hidden layer increased (*M*_8*neurons*_ = 0.275; *M*_16*neurons*_ = 0.221; *M*3_2*neurons*_ = 0.192; *M*64_*neurons*_ = 0.164; *M*_128*neurons*_ = 0.143). Significant main effect also occurred in the ANOVA for the attention parameter, *F*(4, 696) = 15.66, *p* < 0.001, *η*^2^_*p*_ = 0.10. Additional paired t-tests showed that conditions differed significantly from each other depending on the input data (the implemented database contained the values of the 12 oscillators up to 1024; 1792, 2560, 3328 or 4096 ms). As can be seen in Table 2, errors seem to decrease as the database was provided. Yet, after a threshold of 2560 was reached, it appears that further implemented data does not help the system to work better (*M*_1*0*2_3 = 0.196; *M*_1_79_2_ = 0.191; *M*_2_56_*0*_ = 0.201; *M*33_2_8 = 0.203; *M*4_*0*_96 = 0.203). This suggests a threshold effect of attention beyond which more attention does not have much influence. The ANOVA on learning-by-iteration parameter (Number of Epochs) also showed a main effect, *F*(4, 696) = 14.18, *p* < 0.001, *η*^2^_*p*_ = 0.07, and the last ANOVA also found a significant effect for the plasticity parameter (Fahlman offset), *F*(6, 744) = 597.78, *p* < 0.001, *η*^2^_*p*_ = 0.83. Contrasts are exposed Tables 3 and 4 for learning-by-iteration and plasticity parameters, respectively. From a general point of view, it can be say that the errors increased with an increase in the learning by iteration values (*M*_*epoch*1*00*_ = 0.194; *M*_*epoch*2*00*_ = 0.198; *M*_*epoch*_3_*00*_ = 0.199; *M*_*epoch*_4_*00*_ = 0.201; *M*_*epoch00*_ = 0.202), as well with an increase in the plasticity values (*M*_*offset.0*1_ = 0.110; *M*_*offset.0*1_5 = 0.116; *M*_*offset.0*_3 = 0.137; *M*_*offset.0*_6 = 0.189; *M*_*offset.0.1*2_5 = 0.261; *M*_*offset.*2_5 = 0.283; *M*_*offset.0.5*_ = 0.295). It is important to recall that the low *p*-values mentioned above are usual for neural network simulations because the variability around the means is very low.

**Table 1:** Within-subjects effects of the memory parameter (neurons implementation).

Neurons implementation	Neurons implementation	Δ Mean (SE)	95% CI	t value	p_bonf_
8	16	0.054 (0.004)	[0.04; 0.07]	13.405	< 0.001
	32	0.083 (0.004)	[0.07; 0.09]	20.429	< 0.001
	64	0.111 (0.004)	[0.10; 0.12]	27.465	< 0.001
	128	0.132 (0.004)	[0.12; 0.14]	32.646	< 0.001
16	32	0.028 (0.004)	[0.02; 0.04]	7.024	< 0.001
	64	0.057 (0.004)	[0.05; 0.07]	14.060	< 0.001
	128	0.078 (0.004)	[0.07; 0.09]	19.241	< 0.001
32	64	0.028 (0.004)	[0.02; 0.04]	7.036	< 0.001
	128	0.049 (0.004)	[0.04; 0.06]	12.217	< 0.001
64	128	0.021 (0.004)	[0.01; 0.03]	5.181	< 0.001

**Table 2:** Within-subjects effects of the attention parameter (database implementation).

Database implementation	Database implementation	Δ Mean (SE)	95% CI	t value	p_bonf_
1024	1792	0.005 (0.002)	[0.00; 0.01]	0.010	0.043
	2560	− 0.004 (0.002)	[− 0.01; 0.000]	6.757e− 4	0.151
	3328	− 0.007 (0.002)	[− 0.01; − 0.002]	− 0.002	0.002
	4096	− 0.006 (0.002)	[− 0.01; − 0.001]	− 0.001	0.003
1792	2560	− 0.009 (0.002)	[− 0.01; − 0.004]	− 0.004	< 0.001
	3328	− 0.012 (0.002)	[− 0.02; − 0.007]	− 0.007	< 0.001
	4096	− 0.012 (0.002)	[− 0.02; − 0.007]	− 0.007	< 0.001
2560	3328	− 0.002 (0.002)	[− 0.07; 0.003]	0.003	1.000
	4096	− 0.002 (0.002)	[− 0.07; 0.003]	0.003	1.000
3328	4096	0.000 (0.002)	[− 0.05; 0.005]	0.005	1.000

**Table 3:** Within-subjects effects of the learning iteration parameter (epoch modulation).

Epoch implementation	Epoch implementation	Δ Mean (SE)	95% CI	t value	p_bonf_
100	200	− 0.004 (0.001)	[− 0.01; − 0.001]	− 3.091	0.021
	300	− 0.005 (0.001)	[− 0.01; − 0.002]	− 4.203	< 0.001
	400	− 0.007 (0.001)	[− 0.01; − 0.004]	− 5.708	< 0.001
	500	− 0.009 (0.001)	[− 0.012; − 0.005]	− 6.930	< 0.001
200	300	− 0.001 (0.001)	[− 0.005; 0.002]	− 1.112	1.000
	400	− 0.003 (0.001)	[− 0.01; − 0.001]	− 2.616	0.091
	500	− 0.005 (0.001)	[− 0.01; − 0.001]	− 3.839	0.001
300	400	− 0.002 (0.001)	[− 0.005; 0.002]	− 1.504	1.000
	500	− 0.003 (0.001)	[− 0.01; − 0.001]	− 2.726	0.066
400	500	− 0.002 (0.001)	[− 0.005; 0.002]	− 1.222	1.000

**Table 4:** Within-subjects effects of the clock plasticity parameter (Fahlman offset modulation).

Fahlman offset	Fahlman offset	Δ Mean (SE)	95% CI	t value	p_bonf_
0.01	0.015	− 0.005 (0.005)	[− 0.02; 0.01]	− 1.162	1.000
	0.03	− 0.027 (0.005)	[− 0.04; − 0.01]	− 5.708	< 0.001
	0.06	− 0.079 (0.005)	[− 0.09; − 0.06]	− 17.007	< 0.001
	0.125	− 0.151 (0.005)	[− 0.16; − 0.14]	− 32.375	< 0.001
	0.25	− 0.173 (0.005)	[− 0.19; − 0.16]	− 37.073	< 0.001
	0.5	− 0.185 (0.005)	[− 0.20; − 0.17]	− 39.688	< 0.001
0.015	0.03	− 0.021 (0.005)	[− 0.03; − 0.01]	− 4.547	< 0.001
	0.06	− 0.074 (0.005)	[− 0.09; − 0.06]	− 15.845	< 0.001
	0.125	− 0.145 (0.005)	[− 0.16; − 0.13]	− 31.213	< 0.001
	0.25	− 0.167 (0.005)	[− 0.18; − 0.15]	− 35.912	< 0.001
	0.5	− 0.179 (0.005)	[− 0.19; − 0.16]	− 38.526	< 0.001
0.03	0.06	− 0.053 (0.005)	[− 0.07; − 0.04]	− 11.298	< 0.001
	0.125	− 0.124 (0.005)	[− 0.14; − 0.11]	− 26.666	< 0.001
	0.25	− 0.146 (0.005)	[− 0.16; − 0.13]	− 31.365	< 0.001
	0.5	− 0.158 (0.005)	[− 0.17; − 0.14]	− 33.979	< 0.001
0.06	0.125	− 0.072 (0.005)	[− 0.09; − 0.06]	− 15.368	< 0.001
	0.25	− 0.093 (0.005)	[− 0.11; − 0.08]	− 20.067	< 0.001
	0.5	− 0.106 (0.005)	[− 0.12; − 0.09]	− 22.681	< 0.001
0.125	0.25	− 0.022 (0.005)	[− 0.04; − 0.01]	− 4.699	< 0.001
	0.5	− 0.034 (0.005)	[− 0.05; − 0.02]	− 7.313	< 0.001
0.25	0.5	− 0.012 (0.005)	[− 0.03; 0.002]	− 2.614	0.192

In conclusion, as these four parameters related to cognitive and plasticity factors significantly affected the proportion of error in time judgment, we retained the relevant parameters for the elaboration of our Plastic and Cognitive RNN-clock model (CP-RNN-Clock).

## Model 2: the plastic and cognitive RNN inserted within an MLP

### Multilayer perceptron integrating the RNN data

So far, we have modulated four parameters in the RNN to be able to test and model the effect they generated. These variables had an effect on the temporal dispersion coefficient. The objective was then to gather these four variables within the same neural system to test if they matched up with real data. In order to do so, we gathered the results of the launched computation in a Multi-Layer Perceptron (MLP). It would not have been sufficient to use the RNN a second time, because this would have inserted bias a second time via the previous estimate (t-1). The MLP is an artificial neural network similar to that of the RNN, the difference being that recurrent top-down connections are extracted from the model. The architecture thus enables a feedforward mode of operation (as the context layer is no longer present).

### Model construction and stimuli

The totality of the temporal dispersion coefficients paired with each parameters were normalised on a [0; + 1] scale, with the minimum and maximum values being equal to 0 and 1, respectively and inserted as an input layer of a Multi-Layer Perceptron (MLP). Thereby, there were four input variables, namely the number of epochs (*iterative learning parameter*), the amount of information added to the database (*attention parameter*), the plasticity (*plasticity paramete*r), and the number of neurons constituting the hidden layer (*h*_*t*_) (*memory parameter*). There was also a fifth input variable, corresponding to bias. The bias is an additional parameter in Neural Networks which is used to adjust the output along with the weighted sum of the inputs to the neuron. We used the “Regressor” of the python’s neural network toolbox named the “scikit-neural network” to represent this bias. This tool connects the neural network to the given continuous data, treating them as a non-linear regression model. The output of the model was the same output as the initial RNN model (e.g., temporal dispersion coefficient), in order to verify whether or not the new MLP model directly integrating our variables could predict the RNN model.

To sum up, the inputs were the temporal dispersion coefficients paired with the 4 parameters (so 4 inputs; *iterative learning, attention, memory, plasticity*) normalized on a [0; + 1] scale. From all of these initial values, the program had to find a way to re-find each of the dispersion coefficient calculated by the RNN. The output was the dispersion coefficients found based on the modulation of the 4 parameters. The advantage of this process is that it allows the influence of a parameter and its interactions to be treated along a continuous dimension and thus to generalize from the simulated elements. For example, the program can pick a parameter value that lies in the middle of the interval modalities tested by the RNN.

### Computer simulation

**Figure 5:**
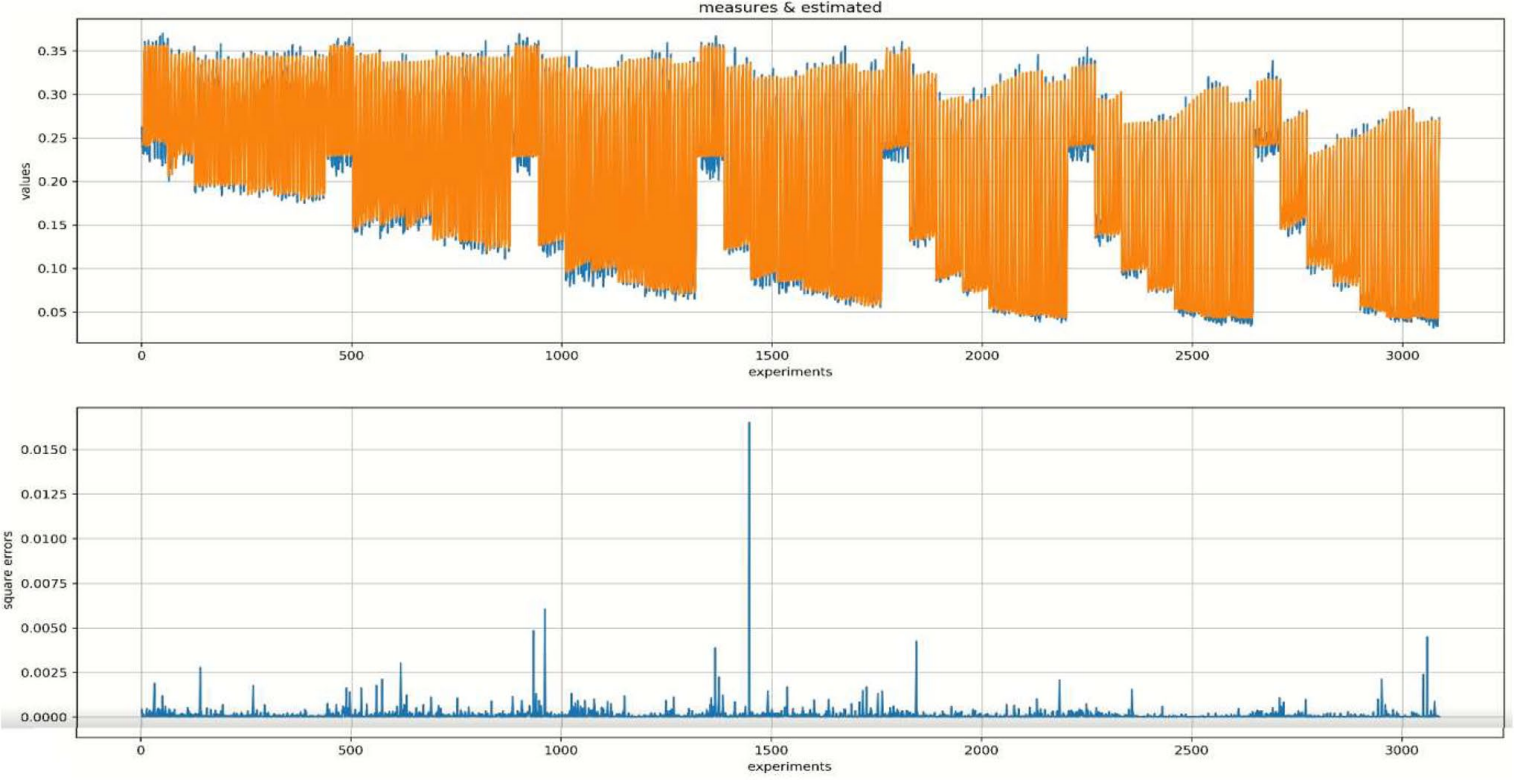
Illustration of data fit with the top panel showing the Euclidean distance values (i.e. the average error produced by the network) observed from the RNN model in blue and estimated by the MLP model in orange with time *t*. The second panel shows the paired square errors*.*

In order to find the best hidden architecture, we ran the model with a range from 8 to 28 neurons constituting the hidden layer. In addition, we tested different combinations with the possibility of multiple hidden layers. Combinations were systematically tested in a linear order, with the constraint for the additional hidden layer of being inferior to the previous one, and being composed of a minimum of three neurons. This specific procedure was chosen in order to use a modeling aspect of the neural network and not a deep-learning one. Our simulations generated a model with 28 neurons made of three hidden layers (**4 – **[**15** – **10** – **3**]** – 1**). This simulation was preserved because it best minimised the mean square error compared to the other architectures tested. Figure 5 show the differences in predictions made by our previous RNN model and the new MLP model directly integrating the four parameters. This specific multi-layer perceptron model is able to determine almost all the temporal dispersions of the RNN model (*R*^2^ = 0.99; LSD = 0.00009; EM = 0.007; EQR = 0.004; EAR = 0.04). We can therefore conclude that our new MLP model, integrating our developmental parameters, is very satisfying, and that it reproduces the properties of the RNN.

## Model 3: fitting the MLP model to participants’ time estimates

### Model construction and real data

**Figure 6:**
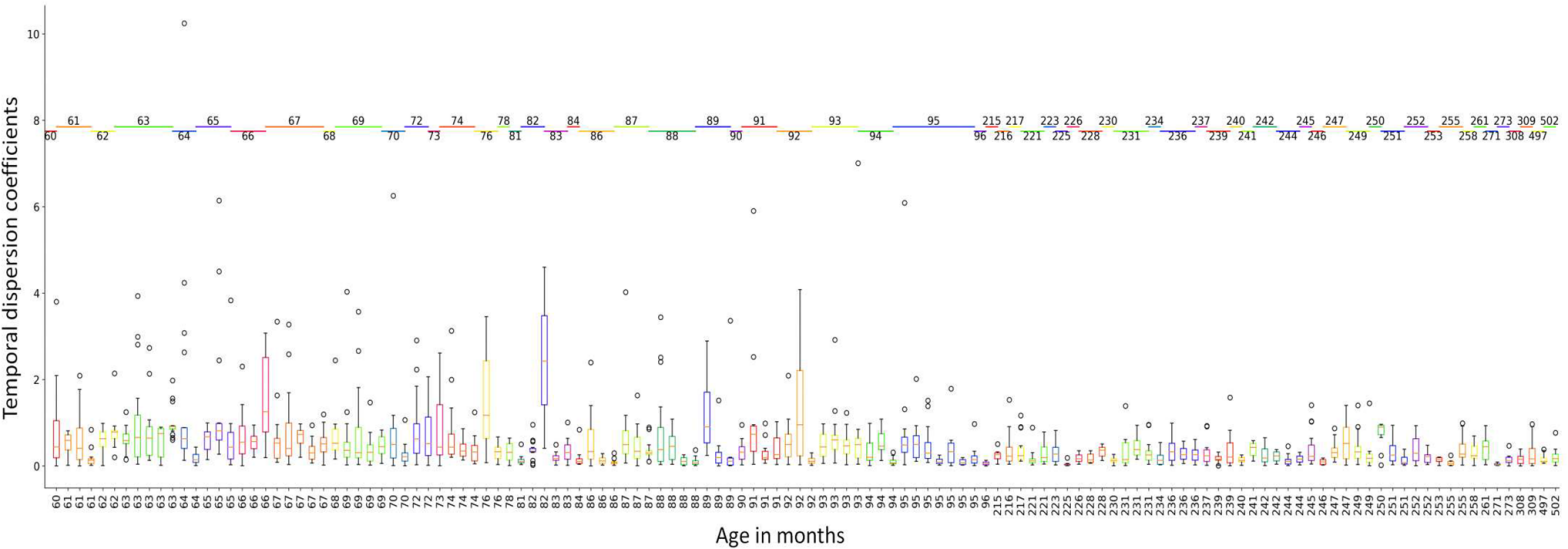
Box-and-whisker of the temporal estimation bias in children and adults with outsider productions represented by circles.

Until this point, we had worked on a coefficient of temporal dispersion calculated from the difference between the prediction of the state of the oscillators and their actual state. Our next goal was to compare the results of our MLP model which integrated all the RNN simulations with real data, to verify its ability to predict real participants’ time estimates according to their inter-individual differences in terms of age and cognitive abilities. The participants’ data were drawn from two different experiments^7,42^ in which they had to reproduce the duration of a light blue square lasting for 3, 6, 9, or 12 s. In total, the data (time estimates) consisted of 2055 temporal reproductions, of which 1358 were made by 192 children aged from 5 to 8 years old (M = 6.62 years, SD = 1.02 years, [5.0; 8.0 years]), and the remaining 697 were made by 116 adults (M = 21.00, SD = 4.17, [17.9; 41.8]). For each temporal reproduction, we calculated the absolute time error made by the participants ((Temporal Reproduction − Temporal Target) / Temporal Target)^2^ to avoid negative and positive values. From these data, 163 productions were rejected (7.93% of the total sample), including 113 children’ productions (8.32% of the child sample) and 50 adults’ productions (7.17% of the adult sample), because they were less than or superior to the 1.5 interquartile range. This made a total of 1892 reliable productions. Figure 6 shows the real temporal coefficient distortion paired with subjects classified by age in months.

For each participant, we also had access to different neuropsychological scores assessing their capacities of attention, short-term memory, working memory, and processing speed. These individual scores were obtained respectively in the “Sky Search” test of the scale of Everyday Attention for Children^43^, the forward and backward scores of the corsi-block tapping test^44^, and the IVT scores measured from the Code A and the Symbol A tests of the Wechsler Intelligent Scale for Children^45^. The choice of these tests was based on the fact that they show heterogeneity in scores among participants, even among adults (see Table 5).

**Table 5:** Mean, Standard Deviation, minimum and maximum of raw scores of different neuropsychological tests.

	Children			Adults		
	Mean	SD	[Min, Max]	Mean	SD	[Min, Max]
Short-term memory	5.55	1.91	[2; 12]	8.91	1.83	[5; 13]
Working memory	4.43	2.18	[0; 9]	8.61	1.73	[4; 12]
Attention	19.23	16.94	[4; 87]	3.81	1.49	[2; 8]
Processing speed	44.28	26.28	[4; 51]	59.2	8.87	[27; 87]

So far, with the MLP integrating the RNN data (**4 – 15 – 10 – 3 – 1**), we have worked on simulated effects to modulate the four parameters. As a reminder, this MLP started with the 4 parameters as input to predict a temporal dispersion (coefficient of dispersion) as output. To analyze the relevance of the model, it is necessary to be able to adapt the four components according to the participants’ neuropsychological scores. This is why a second MLP, which precedes the MLP integrating RNN data, was used to bridge the gap between the individual neuropsychological scores and the 4 components. The idea is to have a sequential processing with a prior MLP, which is the only structure to be dynamic, which precedes the MLP integrating the RNN data, which is fixed and cannot be modulated. To summarize, this final model started with neuropsychological test scores (short-term memory, working memory and processing speed scores) as well as participants’ ages in months as input, to compute the state of the 4 parameters which itself finds the related dispersion coefficient. The learning base was the absolute time error made by participants.

### The results of the fitting

**Figure 7:**
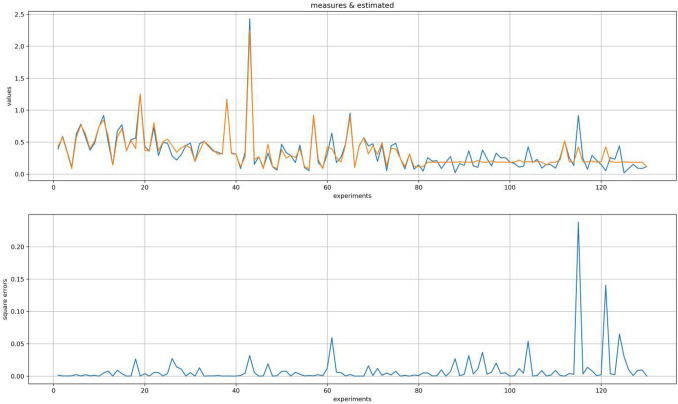
Fitting data with the top panel showing the Euclidean distance values observed from the participants’ productions in blue and estimated by our clock model in orange with time *t*. The second panel shows the paired square errors*.*

To find the best hidden architecture of the prior MLP, which precedes the MLP integrating RNN data, we applied the same procedure as earlier, computing different architecture from 5 to 12 neurons and combining the number of hidden layers. Let’s remind that only the prior MLP was dynamic and could be modulated by the computations, as MLP integrating RNN data was fixed. A model emerged from the computed cases. Figure 7 shows the predictions made by the serial MLPs [5 – 10 – **4 – 15 – 10 – 3 – 1** (The elements in bold constituting the structure of the MLP integrating RNN data). This model is presented here because it obtained the highest level of coefficient of determination when compared with real data (*R*^2^ = 0.90, LSD = 0.009, EM = 0.06, EQR = 1.50, EAR = 0.44).

Through a comparison of means, these final outputs were compared with those of the actual temporal dispersion generated by the participants. Figure 7 shows the one-to-one comparisons between the model’s predictions and actual participant estimates. The results confirmed that the model can predict the state of individuals’ internal clock parameters based on their cognitive abilities and age. From these computed parameters, the internal clock predicted 90% of the variance of the estimation biases of a sample of real subjects comprising both children and adults.

## Discussion

The aim of this study was to propose a new model of internal clock grounded in cognition that we called the Cognitive and Plastic RNN-Clock model (CP-RNN-Clock model). Recently, RNN models have been successfully used to simulate temporal estimates in humans^16,17^. Therefore, we initially tested a Simple RNN clock model, holding the value of each of the oscillations over time (input), to make a judgment on the state of these same oscillators after a certain time (output). The input information was then transformed via a hidden layer, which constantly biases the prediction since it systematically takes into account the combination corresponding to the previous time. Therefore, the prediction of the duration lays on the prediction error (i.e., the cumulative Euclidean distance) between the real state of the oscillators and the prediction for this *t* time. Thereby, the model presents an increase in errors with the passage of time. Our model thus allows the reproduction of the scalar properties of time perception. Indeed, this cumulative Euclidean distance showed that the errors gradually increased over time, therefore testifying to the gradual variance in the model, resulting in further under- or overestimation with the passage of time. Importantly, the increase over time of the errors results from the mechanism of the neural system which we accumulated and does not result from an additional input within the model, as has been done in other models^46^.

The interest of our model is that it is neurobiologically plausible. Indeed, it is based on the rhythmic activity of a bank of oscillators (a set of neurons) that changes state frequently, as proposed in recent neuroscience models on time perception^18,23,47–49^. In addition, the neural frequencies used in our model (32.9–37.2 Hz) are similar to the pattern of neural gamma oscillations observed in humans in the waking state^39,40^. Furthermore, the Simple RNN used in our study includes a loop that is consistent with the cortico-striato-thalamo-cortical loop described in prior imagery studies on timing^50,52^ and in the Striatal Beat Frequency model (SBF) proposed by Mattel and Meck^18^. Consistently with the SBF, our model assumes that cortical oscillators have to be phase-reset, such that they always start from the same fixed state. This is in line with findings showing that phase resetting of ongoing theta oscillations in the medial frontal cortex results in better timing accuracy^53^. Our model also suggests that, once synchronous, the neurons of the cortex begin their rhythmic activity. According to the SBF model, the neurons of the striatum would then play the role of coincidence detector between the state of the various oscillators, since this one receives the entirety of the nervous influx generated simultaneously by the cortex and the thalamus. In other words, the duration would be perceived by neural integration mechanism when the striatal spiny neuron detects the synchronicity of a cortical projection, emerging at the end of the temporal interval. However, for the striatal neuron to retain the pattern of oscillations, it must first have received a sudden dopamine influx. It is this transfer of dopamine that would allow it to go from a Long Term Depression (LTD) where the neuron is inactive, to Long Term Potentiation (LTP) where it can enter into action potential. When a neuron in the striatum recognizes the oscillatory signature associated with the duration of a particular event, the dopamine discharge takes place at the beginning and no longer at the end of the evoked potential. This therefore allows the neuron to enter into an LTP phase, indicating the striatal neuron to start analyzing the oscillatory patterns which reach them until it recognizes the combination (i.e., signature) that corresponds to the end of the duration of the event. Subsequently, information from the striatal spiny neuron is integrated by the basal ganglia and then transferred to the thalamus to generate behavioral expression.

However, unlike the SBF model, in our model, the selected oscillators would never return to a common state during time estimation. Conversely, they would present a progressive desynchronization. This would induce an increase in the error, i.e., the source of the scalar property of time. The strength of our model is therefore that it includes in its functioning the scalar properties of time. Changes in tonic dopamine levels in the brain induced by drugs^54,55^. or high arousal emotion^56,57^ have been shown to accelerate oscillatory activity, thereby producing effects of lengthening time estimates. Theoretically, our model could be able to simulate this other neural phenomenon by generating a faster desynchronization over time. Our model is also neurologically viable because it is based on the use of a RNN to simulate the top-down processing^25^. This is an important feature of the model since the explicit judgment of time is precisely based on top-down processing^28^. Finally, as recent studies demonstrate, the backpropagation exhibited in the model could be a good candidate for neurobiological processes since similar but local training algorithms like equilibrium propagation or energy-based models could actually be very similar to brain-inspired learning rules^58–60^.

Our model was restarted a number of times by changing four parameters of whom 3 were related to cognitive capacities (attention, memory, temporal iterative learning), the fourth being the plasticity of the clock system. We have demonstrated that each of these four parameters had a direct effect on the temporal prediction. Attention was considered in our model via the amount of information held by the model in input. Indeed, lower attention generates a decrease in the relevant information that can be collected in the environment since the information cannot pass the conscious threshold. Meanwhile, increased attention resulted in a decrease in errors. Indeed, our model showed that a higher attention parameter value results in lower errors. Retention capacity in memory was integrated in our model by changing the number of hidden layer neurons. As we have demonstrated, an increase in neurons in the hidden layer leads to a decrease in errors. By changing the Fahlman offset index, we also decreased the temporal dispersion, thereby demonstrating that there could be plasticity effects in the internal clock system. Indeed, as we have explained, a high plasticity setting (characteristic of young children^61^) allows the model to learn faster but can also more frequently give rise to forgetfulness related to a lack of stability, thus resulting in critical prediction errors. Finally, the learning iteration was considered by changing the number of epochs. Increasing the number of epochs increases the number of feedbacks between objective and subjective duration, consequently increasing temporal distortion, and thus having the effect of gradually increasing the time estimation error. We then launched a linear regression on the cumulative Euclidean distance of each of the models launched in order to gain a temporal dispersion coefficient associated with each of the parameter modulations.

The temporal distortion coefficients were then inserted as inputs into a MLP model, that was shown to predict more than 99% of the RNN system estimates. Finally, we compared the results generated by this model with real estimates made by a sample of 192 children aged from 5 to 8 as well as 116 adults. We therefore had to bridge the gap between individual cognitive capacities and the MLP integrating RNN data. To do so, we built a prior MLP, which estimates the 4 parameters based on individual cognitive capacities (short-term memory, working memory and processing speed scores), as well as their ages in months. Let’s remind that only the prior MLP was dynamic, while MLP integrating RNN data was fixed. Mathematical models based on the Scalar Timing Theory^1,62^ have already been developed in order to find the values of tested parameters corresponding to time estimates of each participant^63–65^. However, these models and our neural network model differ both in their purpose and their elaboration. In particular, the estimations of parameters of our model directly resulted from the participants’ individual neuropsychological attention, short-term memory, working memory and processing speed scores, as well as their ages in months. Thus, our model demonstrates how a clock system including cognitive mechanisms, that can be deficient, produces time distortions. Further, our model accounts for more than 90% of the output variance.

The inclusion of these different cognitive variables in our neurological model is important. Indeed, attention has been clearly shown to modulate of neuron synchrony, such as in the somatosensory^66^ or visual cortice^67^. Consequently, any variation in the amount of attention devoted to time would alter the normal oscillation of neurons, thus producing higher distortion over time which is in line with previous studies on time perception. It was also shown that entrained interval can modulate the neural activity peak at the end of the memorized duration^68^. How cognitive activity affects neurological processes is still a mystery. One could nevertheless suggest that most of these variables (plasticity, memory and learning by iteration) acts at this level of neural activity peak, in particular when the striatal neuron receives the cortical projections. These factors could for example contribute to reduce the dopaminergic discharge and noisy the moment when the striatal neuron receives the signature that corresponds to the duration to be estimated.

The originality of our clock model lies mainly in the fact that it is grounded in cognition. As explained above, it is based on the import of clock plasticity and cognitive parameters directly into the operations of the RNN clock model. Including these parameters allowed us to simulate the bias in time estimates and thus to identify the sources of variances due to cognitive and clock-related factors. Consistently with prior studies on time judgments in children with limited cognitive capacities, our model uses neural network mechanisms to explain how decreased attention and memory capacities generate increased temporal errors^3–8^. It also explains how the plasticity of a system and learning iteration increases errors in the temporal memory reference^29,30^. Therefore, our model produced data consistent with developmental studies showing that children can process time at an early age, and that age-related differences in time judgments are mainly due to the development of cognitive capacities^3,4^.

Furthermore, the state of the internal clock’s four parameters were computed based on individual variables (attention, age in months, speed index treatment, short-term memory, and working-memory), suggesting that the weights of interference generated by our plastic and cognitive parameters did not vary by age per se, but maintained the same weight through age, at least from the age of 5 onwards. Indeed, if the variables did not had the same weight in the explanation of the temporal estimation biases in children and adults, it would not have been possible to explain the biases in a common way. Therefore, our model accounts for inter-individual differences in judgment errors. Finally, the model respects the parameter of ordinality, as it knows that *t*_*0*_ is greater than *t*_1_. Indeed, the advantage of an RNN is to intrinsically contain this ordinality given that the snowball effect between the hidden layer and the input layer makes it possible to predict *t*_+1_ on the basis of t, itself predicted by *t*_*−*1_, predicted by *t*_*−*2_…

To the best of our knowledge, this is the first functional clock model based on RNN which is able to explain the inter-individual differences in time judgments related to limited cognitive capacities. However, our Plastic and Cognitive RNN-clock model has some limitations. First, it allows appreciation of the importance of temporal bias, but not its direction (i.e., towards a temporal under- or overestimation). Second, the output variables used in our model were based on a single temporal task, i.e., a temporal reproduction task, and a single sensory modality, i.e., visual stimuli. It is possible that the weight of cognitive capacities would change in the context of other temporal tasks which are more or less cognitively demanding, such as a dual-task paradigm. With other tasks, it could also be necessary to add a decisional rule. In bisection tasks, it has, for example, been shown that the degree of confidence in temporal knowledge can affect the final temporal judgment^69,70^. Similarly, results based on another sensory modality (auditory) could also affect the weights associated to each of the four components tested in our study. It has been shown that temporal judgment of auditory stimuli are less demanding in attention and/or working memory than that of visual stimuli^71–73^. To sum up, it would be beneficial to test our model with various temporal tasks and modalities.

In conclusion, our Cognitive and Plastic RNN-Clock model, grounded in cognition, has succeeded in explaining a significant amount of variances (up to 90%) when its results are compared with 1892 temporal reproduction data collected from children (aged from 5 to 8) and adults (aged from 18 to 42 years). It therefore demonstrates that a lack of cognitive capacities directly interferes in the operation of the clock system which fails, or at least is less able, to predict the state of the oscillators. The originality of our model is that it succeeds in unifying the neurological clock models^17,18,49^ and the models of temporal information processing^1^, based on the scalar expectancy theory^10,74^. Therefore, it considers time judgments by the brain equipped with oscillators and their variations due to the system plasticity and the cognitive capacities that are known to differ between individuals.

### Ethical statements

The data allowing the establishment of the final model are based on protocols which were approved by the research ethical committee IRB-UCA as well as by the ethical standards of the French national research committee (academy) of the French National Education Ministry. This experiment was conducted in accordance with the 1964 Helsinki declaration. The children’s parents and the adults signed a written informed consent to participate in this study.

## Data Availability

The data that support the findings of this study are openly available at: https://osf.io/jyadw/?view_only=bdf4dea174394c2cbca3feaa8326fca9.
